# A Quality Assessment and Evaluation of Credible Online Dietary Resources for Patients with an Ileoanal Pouch

**DOI:** 10.3390/jcm14155348

**Published:** 2025-07-29

**Authors:** Dakota R. Rhys-Jones, Itai Ghersin, Orestis Argyriou, Sue Blackwell, Jasmine Lester, Peter R. Gibson, Emma P. Halmos, Zaid Ardalan, Janindra Warusavitarne, Kapil Sahnan, Jonathan P. Segal, Ailsa Hart, Chu K. Yao

**Affiliations:** 1 Department of Gastroenterology, School of Translational Medicine, Monash University, Melbourne 3004, Australia; peter.gibson@monash.edu (P.R.G.); emma.halmos@monash.edu (E.P.H.); zaid.ardalan@monash.edu (Z.A.); chu.yao@monash.edu (C.K.Y.); 2 Department of Gastroenterology, St Mark’s National Bowel Hospital & Academic Institute, London NW10 7NS, UK; itai.ghersin@nhs.net (I.G.); ailsa.hart@nhs.net (A.H.); 3 Department of Colorectal Surgery, St Mark’s National Bowel Hospital & Academic Institute, London NW10 7NS, UK; orestis.argyriou@nhs.net (O.A.); j.warusavitarne@nhs.net (J.W.); kapil.sahnan@nhs.net (K.S.); 4 Department of Surgery and Cancer, Imperial College London, London W12 0NN, UK; 5 Department of Applied Health Sciences, University of Birmingham, Birmingham B15 2TT, UK; sueblackwell5@gmail.com; 6 Independent Researcher, Adelaide 5000, Australia; jasminelester28@outlook.com; 7 Department of Gastroenterology, Royal Melbourne Hospital, Parkville 3050, Australia; jonathan.segal@mh.org.au; 8 Department of Metabolism, Digestion and Reproduction, Imperial College, London W12 0NN, UK

**Keywords:** ileoanal pouch, online, nutrition, diet

## Abstract

**Background/Objectives**: Patients with an ileoanal pouch change their diet to manage their symptoms and will often resort to the internet for nutrition advice. Currently, no evidence-based dietary guidelines exist to inform online resources. Hence, this study aims to assess the quality of online nutrition information directed towards patients with an ileoanal pouch. **Methods**: A systematic Google search was conducted to identify consumer websites including information on nutrition for those with ileoanal pouches. Quality was assessed using the DISCERN instrument, and the readability of written content was assessed using the Flesch–Kincaid score. A summative content analysis was used to identify the frequency of particular topics. Websites were also assessed against standards from the National Institute for Health and Care Excellence (NICE) framework for shared decision-making support tools. **Results**: A total of 12 websites met the inclusion criteria. Mean total DISCERN scores across all websites are 33 out of 75, indicating that overall, the websites were of poor quality. The mean Flesch–Kincaid score was 57 out of 100, or “fairly difficult” in terms of readability. The main themes according to the content analysis were “general dietary advice for pouch”, “dietary strategies for symptom management”, “addressing risks associated with having a pouch”, and “optimisation of nutritional intake”. Overall, websites did not meet the standards for shared decision-making. **Conclusions**: Online nutrition information for patients with an ileoanal pouch is of poor quality and difficult to understand. There is a need for higher quality online resources for these patients, ideally co-produced with a multidisciplinary team and patient, to provide patients with good quality, understandable, and accessible nutrition information.

## 1. Introduction

Restorative proctocolectomy with ileal pouch–anal anastomosis (IPAA) is one of the main surgical treatments for patients with medically refractory ulcerative colitis (UC) [1] It does, however, have complications, such as pouchitis, that occurs in 60–80% of patients [2,3].

While diet has been implicated in the pathogenesis of pouchitis and other complications, details of the specific components responsible are limited. Despite such poor understanding, patient interest in the use of diets to alter clinical outcomes for their pouch is strong. About 80% of pouch patients have self-reported food intolerances, with the vast majority of patients using diet to control their symptoms such as stool consistency and frequency [4,5]. Depending on the dietary modification employed, these patients could be at a higher risk of nutritional deficiencies, disordered eating practices, and pouch inflammation [6,7,8,9].

When considering the paucity of high-quality dietary studies informing credible, scientifically proven dietary strategies, the internet has become a major source of unregulated nutrition information (or misinformation). One survey highlighted that approximately one third of patients with a UC pouch utilise the internet for dietary advice in preference over dietitians [5]. Access to online unregulated information can have significant implications on a patient’s knowledge regarding their medical condition and treatment options, ultimately affecting decision-making related to their healthcare [10]. Internet search engines such as Google are a typical starting place for healthcare information [11,12] and accounts for more than 65% of all content-related searches online [10]. In addition to traditional search engines, patients are increasingly turning to large language models such as ChatGPT (version 4.0) for health and nutrition information, further complicating the online information landscape [13]. With the wealth of misinformation available online, it is challenging for patients to discern credible dietary advice unless they have strong skills in media and health literacy. The quality and accuracy of online nutrition information across websites in a variety of chronic diseases was found in a systematic review to be poor [14]. Those studies focused on gastrointestinal conditions, such as coeliac disease and irritable bowel syndrome, and had suboptimal nutrition information that was inaccurate and difficult to read. This highlights the importance of understanding the current landscape of online dietary information in order to improve what is accessible to patients and how the information is presented (i.e., readability). To date, there has been no assessment of online consumer information regarding diet for ileoanal pouch patients, particularly from trustworthy sources.

The aim of our study was to examine the quality, readability, shared decision-making, and content of current online information from credible sources regarding nutrition for patients with an ileoanal pouch. This will help to inform organisations to produce better quality online resources so that patients can make dietary choices that best support their health outcomes.

## 2. Materials and Methods

### 2.1. Search Strategy

Google was selected, as it is the most popular search engine. Google incognito mode was used so that personalised search responses were not offered. Searches were performed by two authors (DRJ and IG). They were performed from different countries (Australia and the UK) in order to minimise location bias. The keywords used in the search were “diet and ileoanal pouch”, “diet and J pouch”, “nutrition and ileoanal pouch”, and “nutrition and J pouch”. Searches were run separately for each keyword to ensure that no sources were missed. Only the first three pages of results for each search were screened, as most people typically stop after the first page [15].

### 2.2. Eligibility Criteria

Consumer information materials that are written in the English language on nutrition in ileoanal pouch patients and produced by a trustworthy source defined as a charity or healthcare organisation, professional association, or a private healthcare provider were included. All types of information resources were included, ranging from a single webpage and blog-style posts to patient leaflets.

Exclusion criteria were patient-to-patient sources sharing experiential information. Any sources that appeared only as paid for Google ads were also excluded, as paid-for-search results are temporary and may not appear if the search is replicated later.

### 2.3. Data Extraction and Analysis

Data were extracted by DRJ and IG from websites available as of 27 June 2024 into Microsoft Excel [Microsoft, Redmond, WA, USA]. Basic characteristics regarding each website including name or organisation, type of document, country of origin, type of organisation, and general notes related to the content of each website were documented. All information resources were assessed using validated tools for the quality of its content (DISCERN) and readability (Flesch–Kincaid reading ease score). Fleiss Kappa was determined to examine the inter-rater agreement of DISCERN scores using Excel [Microsoft, Redmond, WA, USA]. All websites were also scored against the National Institute for Health and Care Excellence (NICE) standards framework for shared decision-making, which determines whether it supported shared decision-making [16]. Full texts of the sources were imported into NVivo 14 [Lumivero, Denver, CO, USA], which was used for content analysis.

### 2.4. Content Quality Analysis

The DISCERN tool is a validated instrument that determines the quality of written consumer health information and was designed to be used by health professionals and patients [17]. The questionnaire has three sections assessing reliability, quality, and overall ratings via a 5-point Likert scale across 16 questions, with 1 demonstrating strong disagreement and 5 indicating strong agreement. It does not require specific training for use. One question was omitted—“Does it describe what would happen if no treatment is used?”—because it was not deemed relevant in the context of diet. The total DISCERN score was adjusted accordingly. Importantly, the DISCERN instrument does not judge scientific accuracy; rather, it evaluates the reliability, transparency, and quality of information presented to consumers to make informed healthcare decisions.

As the DISCERN score is based on the reviewer’s judgement and is thus prone to reviewer bias, the sources were analysed independently by two assessors from different disciplines (dietitian DRJ and gastroenterologist IG) who were blinded to each other’s assessment. Additionally, to capture the patient perspective and evaluate consistency across raters, 50% of the resources were selected at random and scored by a third independent assessor (JL, patient). Discrepancies were defined as differences in score of ≥2 and were resolved via a group consensus. The mean for each question across all websites was then calculated and summed to obtain a total DISCERN score, which was adjusted to reflect the omission of one question. An adapted scoring system based off previous studies was implemented for the total quality of the website—“excellent” as 60–75 (≥80% score), “good” as 49–59 (65–79%), “fair” as 38–48 (51–64%), “poor” as 28–37 (37–50%), and “very poor” as 15–27 (20–36%) [18,19].

### 2.5. Readability of Written Information

Readability was assessed to identify how accessible the websites are to individuals of lower literacy levels. It was scored by the Flesch–Kincaid reading ease score, using the readability function in Microsoft Word [Microsoft Corporation, Windows 10, Redmond, WA, USA] [20]. The Flesch–Kincaid score uses a formula based on the average sentence length in words and the average word length in characters and gives the text a numerical value of 0–100. The higher the score, the greater the readability. The ease of readability was also assessed categorically from “very difficult” (0–30), to “very easy” (90–100). For benchmarking purposes, a score above 60 for written health information is recommended, because this reflects the average readability of a ~13-year-old and would suggest a wider audience is reached [21], whereas the converse applies for scores below 60.

### 2.6. Content Analysis

A summative content analysis was performed using NVivo 14 software [Lumivero, Denver, CO, USA] to evaluate the frequency of particular topics on each website [22]. Themes were identified before and during analysis for each information resource. Themes were initially independently coded by DRJ and then discussed, refined, and grouped together to form themes and subthemes which were applied to the remaining sources by DRJ. Only sections on diet were included in the final analysis.

### 2.7. Shared Decision-Making Analysis

Websites were assessed according to the NICE standards framework for shared decision-making by two team members (DRJ and OA). This framework helps healthcare services to create good quality “patient decision aids”—tools that are designed to provide evidence-based information about healthcare decisions [16]. Each “essential content standard” within the framework was scored as “yes”, “no”, or “partial”. It should be noted that even though the identified sources were not formal shared decision-making tools, patients use these websites as sources of information when discussing dietary treatment options with healthcare professionals. Thus, assessment against the NICE standards was deemed appropriate. Any disagreements were resolved by consensus.

## 3. Results

A total of 12 consumer websites met the inclusion criteria. Table 1 details the characteristics of each source of consumer dietary information. Seven of the twelve websites were online information leaflets, while the rest were webpages or blogs. Four were government healthcare organisations, three were academic institutions, three were foundations/associations related to IBD, and two were private clinics. Of the five websites from the UK, three had similar content produced by a UK-based hospital across each of the websites. One website (United Ostomy Association, Biddeford, ME, USA) was an extensive document related to the whole surgical procedure in the formation of an ileoanal pouch, and only the section relevant to diet was included.

### 3.1. Content Quality

**Figure 1:**
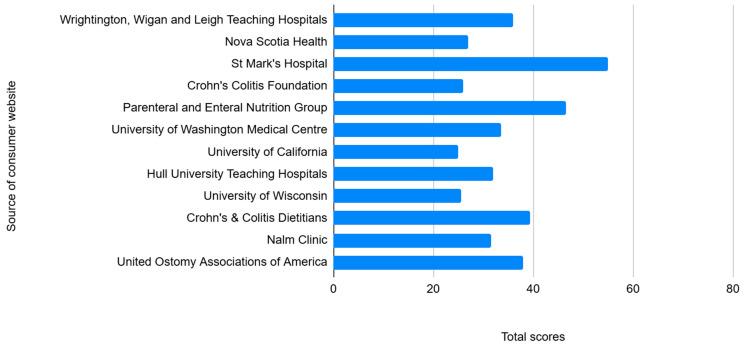
Overall quality scores for consumer websites assessed using the DISCERN tool providing information about diet for patients with an ileoanal pouch.

Figure 1 outlines the total scores for each website using the DISCERN instrument. Of the total possible score of 75, the mean overall score across all websites was 33 (range 25–55), indicating that the websites were of poor quality. The majority of the websites (75%) scored below 38 or poorly/very poorly, and none had scores above 60, indicating “excellent” quality. St. Mark’s Hospital’s website scored the highest at 55, and the University of California scored the lowest with 25.

The inter-rater agreement using Fleiss’ kappa between clinicians (DRJ and IG) and the patient (JL) was a score of 0.34 or a fair agreement. Discrepancies were discussed via group consensus. In most questions that had a discrepancy, quality was rated higher by the patient representative than by clinicians.

### 3.2. Readability

The Flesch–Kincaid scores for each website are embedded within Table 2, as the shared decision-making tool (below) also utilises this score. The mean score across all websites was 56.6 out of a total possible score of 100 (range 42.4–73.5). Five of the twelve websites (42%) scored above 60. The website with highest readability score was Nova Scotia Health with a score of 73.5, and the lowest was Nalm Clinic with a score of 42.4.

### 3.3. Summative Content Analysis

A summative content analysis identified four broad themes of nutrition information that were conveyed by the information resources—"general dietary advice for pouch”, “dietary strategies for symptom management”, “addressing risks associated with having a pouch”, and “optimisation of nutritional intake”. Table 3 details the themes and subthemes.

- General dietary advice for pouch: Recommendations of specific food to be consumed were most commonly mentioned (*n* = 10 websites), where some explained reasons such as “protein-containing foods for repair of muscle” and others just provided lists of foods to include. Foods to avoid (*n* = 9) also varied and included high-sugar foods and spicy foods. Eating styles (*n* = 9), such as the timing of meals and specific nutrients to consume (*n* = 9), were the next most common and also varied in specific details. The implementation of a pre-surgical diet using the enhanced recovery after surgery (ERAS) protocol was the least common (*n* = 1).
- Dietary strategies for symptom management: Most websites provided advice on specific foods to decrease output (*n* = 8) followed by increasing output (*n* = 7), reducing anal irritation (*n* = 7), increasing wind (*n* = 6), passing undigested food (*n* = 4), bloating (*n* = 4), increasing stool odour (*n* = 4), decreasing stool odour (*n* = 1), loose stools (*n* = 3), and increasing urgency (*n* = 2). There were duplicates in this, with the same table being used across the St. Mark’s-related documents. Most listed individual foods, beverages, and/or ingredients, while some included foods with multiple ingredients, such as suet pudding and coleslaw.
- Addressing risks associated with having a pouch: Dehydration was the most commonly mentioned risk (*n* = 9), followed by pouchitis (*n* = 5), bowel obstructions, (*n* = 3), incontinence or leakage (*n* = 2), and (abnormal) bile acid malabsorption (*n* = 1).
- Optimisation of nutritional intake: Websites focused on specific nutrients to consume or increase in pouches (*n* = 9), as well as nutrients of concern specific to pouches (*n* = 5).

### 3.4. Shared Decision-Making

Table 2 shows the outcomes of each online source of information assessed against the standards framework for shared decision-making by NICE for patient decision aids. All webpages, except for the Crohn’s Colitis Foundation, met at least some of the criteria for the “health condition and available options”, which regards presenting detailed information about treatment options to ensure that an informed and unbiased decision can be made. The NICE framework suggests that patient decision aids should encourage patients to discuss their priorities and decisions with people close to them, under the “support for the person’s values, circumstance and preferences” criteria, which no website scored a positive result in. Only two websites clearly had consumer involvement and co-design, but their contribution was unclear. No website scored positively for risks and benefits—where a neutral presentation of risks and benefits is supposed to be presented using easy-to-understand numerical data. For all websites, when numerical data was presented, it was not in formats recommended by the guidelines.

## 4. Discussion

Patients with inflammatory bowel disease are increasingly accessing the internet for quality nutrition information to make informed choices about their diet/treatment. Alongside this is a demand for credible, easy-to-read, and consistent nutrition information from trustworthy websites. In this study, we examined the current state of online nutrition information available to patients living with an ileoanal pouch regarding these quality constructs. We found the available information is mostly of poor quality. Furthermore, it does not meet the recommendations for ease of readability, limiting the reach of patients who will understand these pages. There are currently no shared decision-making tools or patient decision aids available regarding nutrition for ileoanal pouch patients. Therefore, it is likely that patients will use the online sources identified to make nutritional decisions. When assessing these online sources against shared decision-making criteria, most of them scored “partial”, reflecting their biassed nature.

Poor-quality online information is not confined to diet in ileoanal pouch patients. Unfortunately, studies examining online information on the surgical treatment of colorectal diseases, such as colorectal cancer [23], inflammatory bowel diseases [24,25,26], and parastomal hernias [27], have also demonstrated information that is low quality and of inadequate readability. In a study of inflammatory bowel disease patients using the internet to access health information, 84% of patients wished that their gastroenterologists would direct them to trustworthy websites and 65% were willing to pay for reliable information [28]. Additionally, studies have consistently found nutrition information online to be of low quality and difficult to understand [19,29,30,31]. Despite including websites from trusted sources, such as major hospital services, no consumer website scored excellent for quality in our study, and only one was considered easy to understand. If educational materials from trusted sources, such as healthcare institutions, are not understood by patients, they may turn to other less credible sources for nutrition information, such as social media accounts. Although the data to support online nutrition information from good-quality dietary trials in ileoanal pouches are lacking, this does not justify poor quality or hard-to-understand websites from trusted organisations. Thus, although there is a demand for good-quality online healthcare information from patients, the results from our study indicate problems with the output of high-quality online nutrition informational resources in colorectal surgery, gastroenterology, and nutrition that present a major gap that needs addressing.

Interestingly, while both healthcare professionals (DRJ, a dietitian, and IG, a gastroenterologist) graded informational sources similarly using the DISCERN tool, there were notable discrepancies between the grades given by the medical professionals and those given by our patient representative. For example, the patient (JL) rated DISCERN scores consistently higher for various questions compared with those from the researchers. One of the questions focuses on the relevance of the content from a patient perspective—this clearly can only be hypothesised if the person answering this question is not a patient. This is an important consideration when these websites are designed to target patients. The subjectivity inherent in the DISCERN tool means that interpretations vary based on life experience and individual relevance. This underscores the importance of patient perspectives when using this tool and in the development of online health resources.

The content analysis demonstrated marked variability in topics between sources. The suggestions regarding foods to include or avoid were inconsistent. For example, while most listed individual food items that did follow some patterns, such as high-FODMAP ingredients like onion/garlic causing gas, some listed dishes contained mixed ingredients such as “suet pudding”, “Chinese food”, or “juice”. Avoiding entire cuisines, dishes and all juices entirely may be recommendations based on experience but may add to the confusion these patients experience. It is worth noting that while most websites focused on adequate hydration and short-term post-operative diet, long-term diet was rarely discussed. This reflects a significant unmet need for patients. The provision of basic information on long-term healthy eating equips the patient to make informed decisions rather than a “trial-and-error” approach, leading to unnecessary restrictions. Additionally, most resources did not discuss potential vitamin or mineral deficiencies and the need for supplementation, another important aspect that requires careful attention in pouch patients [32,33].

Patient decision aids are tools used in healthcare to support shared decision-making between patients and healthcare professionals. The NICE shared decision-making framework focuses on the creation of patient decision aids that increase patient knowledge in an area while considering their personal values and circumstances. There are studies using patient decision aids in nutritional oncology and pregnancy in IBD but none that currently exist specifically for IBD [33,34,35,36]. Thus, patients rely on online resources that, as we found, have limited input from patients and do not meet criteria that support the individual’s values, circumstances, and preferences. Given that patients are making decisions related to their diet every day, healthcare institutions creating these documents should consult the NICE shared decision-making guidelines when creating resources for patients with an ileoanal pouch.

There are limitations to this study. It included only English-language sources. There may be higher quality nutrition information material in other languages which were not included in our analysis. Additionally, there are probably locally produced printed information resources in outpatient departments, which may form a significant source of information transfer from clinician to patient, which cannot be found online. This study was also confined to written online information from Google, not from YouTube or other social media platforms; however, written, audio, and visual tools would require different quality assessment tools. Additionally, omitting one question from the DISCERN tool may limit direct comparability to other studies using the full 16-item instrument. However, presenting overall scores as percentages helps preserves comparability.

## 5. Conclusions

Current online information regarding nutrition for patients with an ileoanal pouch is of insufficient quality and inadequate readability. Organisations should ensure that the dietary information on their websites are not only evidence-based but clear, understandable, and readable so that patients can make better informed dietary choices.

## Figures and Tables

**Table 1:** Characteristics of consumer websites providing dietary information on ileoanal pouch management.

Name of Organisation	URL	Type of Organisation	City and Country of Origin	Format of Diet Information
Wrightington, Wigan and Leigh Teaching Hospitals	https://www.wwl.nhs.uk/media/.leaflets/5ffeb6edcf7e70.74545301.pdf (accessed 27 Jun 2024)	Government healthcare	Wigan, UK	Four-page patient leaflet
Nova Scotia Health	https://www.nshealth.ca/patient-education-resources/0555 (accessed 27 Jun 2024)	Government healthcare	Halifax, Canada	Eight-page leaflet
St. Mark’s Hospital	https://www.stmarkshospital.nhs.uk/wp-content/uploads/2014/05/Healthy-eating-for-people-with-internal-pouches.pdf (accessed 27 Jun 2024)	Government healthcare	London, UK	Ten-page leaflet
Crohn’s Colitis Foundation	https://www.crohnscolitisfoundation.org/patientsandcaregivers/diet-and-nutrition/surgery-and-nutrition/j-pouch-surgery-nutrition (accessed 27 Jun 2024)	Foundations/associations	New York City, USA	Single online page
Parenteral and Enteral Nutrition Group	https://www.peng.org.uk/pdfs/diet-sheets/internal-pouches.pdf (accessed 27 Jun 2024)	Foundations/associations	Birmingham, UK	Six-page patient leaflet
University of Washington Medical Centre	https://healthonline.washington.edu/sites/default/files/record_pdfs/J-Pouch_Nutritional_Guidelines_9_09.pdf (accessed 27 Jun 2024)	Academic institution	Seattle, USA	Two-page leaflet
University of California	https://www.ucsfhealth.org/education/special-concerns-for-people-with-j-pouches (accessed 27 Jun 2024)	Academic institution	Oakland, USA	Single online page with various issues related to surgery, including a section on diet
Hull University Teaching Hospitals	https://www.hey.nhs.uk/patient-leaflet/eating-with-an-ileoanal-pouch/#:~:text=Eating%20small%20amounts%20more%20frequently,as%20Quorn%2C%20Tofu%20and%20tempeh (accessed 27 Jun 2024)	Government healthcare	Hull, UK	Single online page
University of Wisconsin	https://patient.uwhealth.org/healthfacts/355 (accessed 27 Jun 2024)	Academic institution	Madison, USA	Two-page leaflet
Crohn’s & Colitis Dietitians	https://crohnsandcolitisdietitians.com/j-pouch-surgery-what-to-eat-the-nutritional-implications/ (accessed 27 Jun 2024)	Private clinic	USA	Blog-style page
Nalm Clinic	https://nalmclinic.com/blog-1/2022/2/14/is-diet-important-with-a-j-pouch (accessed 27 Jun 2024)	Private clinic	London, UK	Blog-style page
United Ostomy Associations of America	https://www.ostomy.org/wp-content/uploads/2024/03/IleoanalReservoir_J-Pouch-Guide.pdf (accessed 27 Jun 2024)	Foundations/associations	Biddeford, USA	Twenty-nine-page patient leaflet about an internal reservoir—including a section on diet

**Table 2:** Comparison of website pages to the standards framework for shared decision-making by the National Institute of Care and Excellence.

Source	Health Condition, Decision, and Available Options	Details of Available Options	Support for Person’s Values, Circumstances, and Preferences	Use of Language and Numbers/Flesch–Kincaid (Readability) Score		Formats and Availability	Evidence Sources	Patient Involvement and Co-Production	Risks and Benefits	Review Cycle and Declaration of Interests
Wrightington, Wigan and Leigh Teaching Hospitals	Partial	Yes	No	Language easy to understand but no figures or numbers. No diagrams or numbers, but pictures and tables to break up text.	62.6	Yes—“this leaflet is also available in audio, large print, Braille and other languages upon request”	No	No	Partial	Partial
Nova Scotia Health	Partial	Yes	No	Language easy to understand. No diagrams or numbers, but pictures and tables to break up text.	73.5	Yes—website with PDF print out available with links to online pamphlets/resources	No	No	No	Partial
St. Mark’s Hospital	Partial	Partial	No	Fairly easy to understand. However, low Flesch–Kincaid score. No diagrams or numbers, but pictures and tables to break up text.	43.1	Yes—online website for PDF print out. Mentions discussing decision-making with HCP	Partial	Yes	Partial	Yes
Crohn’s Colitis Foundation	No	No	No	Fairly easy to understand language. However, low Flesch–Kincaid. No diagrams, numbers, or tables.	47.6	Single online page	Yes	No	No	No
Parenteral and Enteral Nutrition Group	Partial	Yes	No	Fairly easy to understand. However, low Flesch–Kincaid score. No diagrams or numbers, but text is broken up by a table.	49.7	Yes—six-page website for PDF print out	Partial	No	Partial	No
University of Washington Medical Centre	Partial	Partial	No	Language easy to understand. All of the text in dot points and a table.	70.6	Yes, two-page online website PDF for print out	No	No	No	Partial
University of California	Yes	Partial	No	Fairly easy to understand language with Flesch Kincaid score. No use of diagrams, visuals, numbers, or tables.	59.3	Single online page	Partial	No	Yes	No
Hull University Teaching Hospitals	Partial	Partial	No	Language easy to understand. Most of the text in dot points, also a table.	64.4	Yes—online site is translatable into seven languages	No	No	Partial	Partial
University of Wisconsin	Partial	Partial	No	Language easy to understand. No figures, numbers, or tables.	70.4	Double page online PDF for print	No	No	Partial	No
Crohn’s & Colitis Dietitians	Yes	Partial	No	Fairly easy to understand language but does get technical when discussing studies. Flesch–Kincaid score low. No use of diagrams, pictures, or numbers.	45.4	Online webpage	Partial	No	Partial	Partial
Nalm Clinic	No	No	No	Fairly easy to understand language; however, does get technical when discussing studies. Flesch–Kincaid score low. No use of diagrams, pictures, or numbers.	42.4	Online webpage	Partial	No	Partial	No
United Ostomy Associations of America	Partial	Partial	No	Fairly easy to understand language but does get technical in certain sections. Flesch–Kincaid score low. Use of diagrams and tables to support text.	49.7	Online booklet and guidebook with PDF print out available	Yes	Yes	Partial	Yes

**Table 3:** Content analysis themes and subthemes.

Category	Codes	No. of Websites
General dietary advice for pouch	Pre-operative nutrition	1
	Initial post-operative diet	7
	Eating styles	9
	Foods to include	10
	Foods to avoid	9
	Long-term diet	3
	Use of supplements	4
Diet strategies for symptom management	Decrease output	8
	Increase output	7
	Anal irritation	7
	Increase wind	6
	Passing undigested food	4
	Bloating	4
	Increase stool odour	4
	Decrease stool odour	1
	Loose stools	3
	Increased urgency	2
Addressing risks associated with having a pouch	Dehydration	9
	Pouchitis	5
	Bowel obstruction	3
	Bile acid malabsorption	1
	Incontinence or leakage	2
Optimisation of nutritional intake	Nutrients to consume/increase specific to pouches	9
	Nutrients of concern specific to pouches	5

## Data Availability

The data underlying this study are available in the published article.
